# Supplementary material for: Eliminating yellow fever epidemics in Africa: Vaccine demand forecast and impact modelling
Source: PLoS Negl Trop Dis. 2020 May 7;14(5):e0008304. doi: 10.1371/journal.pntd.0008304 (PMC7237041; doi:10.1371/journal.pntd.0008304)
Supplement: S1 Appendix — (DOCX) [file pntd.0008304.s002.docx]

**Eliminating yellow fever epidemics in Africa: vaccine demand forecast and impact modelling**

**Short title :** Modelling the Elimination of Yellow Fever epidemics in Africa

**S1 Appendix : Model description**

Kévin Jean^1,2,3^*, Arran Hamlet^3^, Justus Benzler^4,5^, Laurence Cibrelus^4^, Katy A. M. Gaythorpe^3^, Amadou Sall^6^, Neil M. Ferguson^3^, Tini Garske^3^.

1. Laboratoire MESuRS, Conservatoire National des Arts et Métiers, Paris, France
2. Unité PACRI, Institut Pasteur, Conservatoire National des Arts et Métiers, Paris, France
3. MRC Centre for Global Infectious Disease Analysis, Department of Infectious Disease Epidemiology, Imperial College, London, UK
4. Infectious Hazard Management, World Health Organization, Geneva, Switzerland.
5. Robert Koch Institut, Berlin, Germany
6. Arbovirus and viral haemorrhagic fever unit, Institut Pasteur de Dakar, Dakar, Senegal

* Correspondance to:

Kévin Jean

Laboratoire MESuRS, Conservatoire National des Arts et Métiers, 292 rue Saint Martin, 75003, Paris, France

kevin.jean@lecnam.net

Index table

[1. Datasets 27](#_Toc529350703)

[1.1. Yellow Fever occurrence 27](#_Toc529350704)

[1.2. Demographic data 27](#_Toc529350705)

[1.3. Vaccination data 28](#_Toc529350706)

[1.4. Serological surveys 28](#_Toc529350707)

[1.1. Environmental and geographical datasets 29](#_Toc529350708)

[2. Model structure 29](#_Toc529350709)

[2.1. Generalized linear model for the presence/absence of any yellow fever report 30](#_Toc529350710)

[2.2. Estimating the per-case probability of report 31](#_Toc529350711)

[2.3. Fitting serological data 31](#_Toc529350712)

[2.3.1. FOI model 32](#_Toc529350713)

[2.3.2. $R0$ model 32](#_Toc529350714)

[2.4. Extrapolating the transmission parameters 35](#_Toc529350715)

[2.5. Estimating the total number of infections from transmission intensity 35](#_Toc529350716)

[3. Model fitting 35](#_Toc529350717)

[4. Burden estimates 36](#_Toc529350718)

[5. Vaccine impact estimates 37](#_Toc529350719)

The Yellow Fever Burden model has been developed to assess the burden of Yellow Fever at the first subnational level in the endemic zone of Africa, relying of a variety of data. The first description of the model was published in 2014 [9].

Different geographic scales are used in this model, mostly the first (hereafter called province) and the second subnational level (hereafter called district). Some model components (e.g. population estimates) or intermediate outputs (e.g. vaccination coverage) are estimated at the district level, whereas transmission intensity and burden estimates are provided at the province level.

Different transmission cycles for yellow fever co-exist. In the sylvatic cycle, human infections occur as spillover events from the wildlife reservoir (non-human primates). In the urban cycle, transmission arises due to human-to-human transmission mediated by the urban vector of the disease, *Aedes aegypti*. The sylvatic and urban transmission cycles are captured by a static and dynamic version of the model respectively. In the static model, transmission intensity is parametrized in terms of a static force of infection, while in the dynamic model it is parametrized by a static value of R0, using the steady-state equations of the classical SIR model framework. This is then converted to a dynamically varying force of infection, depending on population immunity caused by natural infection and vaccination. The models mainly differ in the way they account for vaccination. In the static version of the model, vaccination only protects vaccinated individuals. In the dynamic version of the model, unvaccinated individuals are protected to some degree due to the reduction of transmission in the human population as a consequence of the achieved vaccination coverage. We would expect herd effects to result in a larger impact of vaccination in the urban cycle than in the sylvatic cycle.

# Datasets

## Yellow Fever occurrence

We created a database of location and dates of yellow fever reports over a 30-year period (1984 to 2013) compiling data of two kinds: outbreak reports and individual lab-confirmed cases. Outbreak reports were compiled from the Weekly Epidemiological Reports (WER), the WHO Diseases Outbreak News (DON), and the published literature, and located at the province level. Individual confirmed cases were provided by the Yellow Fever Surveillance Database (YFSD), a surveillance database established by the African Regional Office of WHO. This surveillance system covers 21 countries in West and Central Africa and records suspected cases using a deliberately broad case definition (based on fever and jaundice). Suspected cases are then laboratory confirmed or discarded accounting for differential diagnosis and vaccination history. Outbreak records and lab-confirmed cases are combined in an overall dataset recording the presence or absence of any reported yellow fever event between 1984 and 2013 at the province level.

Due to the broad case definition used, the majority of suspected cases reported in the YFSD are eventually attributed to causes other than yellow fever. Thus, the national annual rate of suspected cases can be better interpreted as a metric of the national surveillance effort. The annual number of suspected cases is thus aggregated by country and divided by the total population size in order to be used as a metric of the surveillance quality.

## Demographic data

A dataset of population sizes and age structures at the province level was created, covering the 1940-2100 time period. For each country, population size and age structure were compiled from the UN World Population Prospects (WPPs) [35]. The 5-year age band from UN WPPs dataset were disaggregated into annual birth cohorts as previously described [36].

These estimates were combined with spatial population distribution from Landscan 2014 version [37] in order to estimate population sizes at the 2^nd^ (district) sub-national level. Landscan provides population size estimates for pixels of 1/120-degree latitude and longitude (approximatively 1 square-km at equator). Population sizes were aggregated at the district level and the proportion of the total country population living in each district was estimated. District population sizes were then calculated by applying these proportions to the UN WPPs country-level population sizes. In the absence of more detailed datasets, we assumed that age structure and population growth were homogeneous within each country, and that the population spatial distribution did not change over time. We further assumed that age-specific death rates are not affected by yellow fever infection or vaccination as the yellow fever related mortality is already included in the overall mortality in the demographic projections we use.

By combining UN WPPs data and spatially explicit demographic data, for each province and each year$t$, we were able to determine the total number of people $P\left( a,t \right)$ as well as the fraction of the population $p(a,t)$ aged $a$ at the year $t$.

## Vaccination data

We used data from historical vaccination activities dating back to the 1940s [38], reactive campaigns, recent preventive mass-vaccination campaigns [39], and routine infant vaccination [40] in order to create a vaccination activities dataset. Information on vaccination activities was compiled into a dataset of age-specific population immunity at the district level, taking into account the extent of each campaign, in terms of number of doses or coverage achieved, the location as well as the demographics of the targeted populations. The resulting list of vaccination activities has been compared with data from the International Coordinating Group (ICG) on vaccine provision for yellow fever of the WHO and discrepancies were corrected. The achieved vaccination coverage was then tracked by age and district through time using a demographic model informed by the population datasets in order to estimate the province-, age- and time-dependent vaccination coverage $v_{iat}$. In order to account for imperfect vaccine efficacy, this coverage dataset was further multiplied by an efficacy factor based on a meta-analysis on serological response associated with yellow fever vaccination [25].

The detailed method for estimating vaccination coverage has been presented elsewhere [14], and a visualization tool for the past, present and future population immunity across the endemic zone in Africa is available at https://polici.shinyapps.io/yellow_fever_africa/.

## Serological surveys

The model was calibrated using serological surveys conducted in 11 African countries (Figure 1). The original yellow fever burden model as described in 2014 was calibrated using six serological surveys conducted between 1985 and 2009 and identified by a literature review [41–46]. Each serological survey was attributed to one or several provinces based on the geographical information available in the corresponding article. Only surveys that had samples tested for yellow fever virus specific neutralizing antibodies were considered. Serological surveys that were part of outbreak investigation were excluded, as they would be expected to rather measure the outbreak-specific attack rate rather than the average transmission intensity over a long time period. More recently, as part of the WHO yellow fever risk assessment process, serological surveys based on yellow fever virus specific neutralizing assays have been performed in six East African countries. Results of these surveys are aggregated by ecological zone(regions sharing similar ecological characteristics, as defined by national authorities), with one or more survey sites per ecological zone. Based on the localization of the survey sites, for each ecological zone (as defined within the WHO risk assessment process), the results were attributed to one or several provinces.

## Environmental and geographical datasets

Remote-sensing data on Enhanced Vegetation Index (EVI, averaged over the 2003-2006-time period) [47] and land cover category indicating a mosaic of cropland and natural vegetation were used [47,48]. These data were combined with geographical information on administrative areas provided by the GADM project [49]. Variables were available with different spatial resolutions and were aggregated to the province level by calculating the population-weighted mean of each pixel falling into a province. This was done to obtain values representative of locations were human population are concentrated.

# Model structure

A schematic overview of the model is presented in Figure 1:


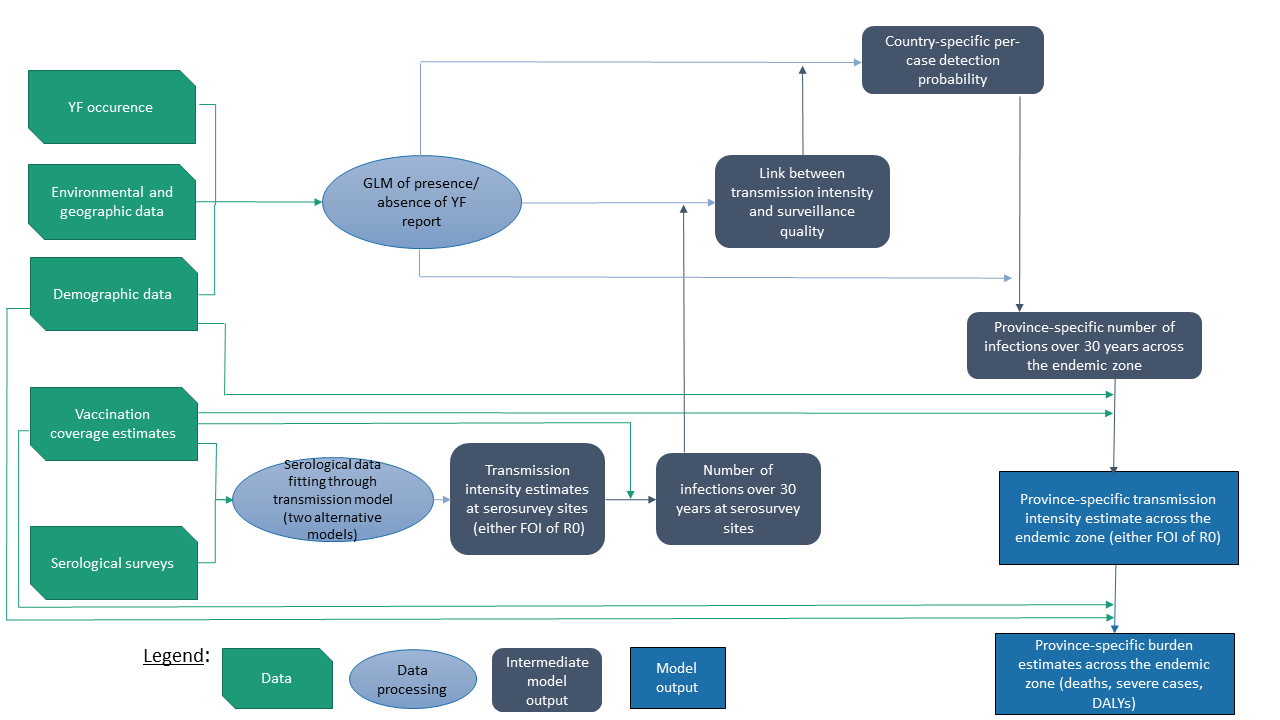


Appendix 1 - Figure 1: Schematic overview of the Yellow Fever Burden model.

The notation used for the model description is defined in Table 1.

Appendix 1 - Table 1: Definition of the notation used in the model description.

| Notation | Definition |
| --- | --- |
| Generalized linear model | |
| $\beta$ | Parameter vector for the GLM for the presence/absence of any yellow fever report |
| $q$ | Prediction of the GLM for the presence/absence of any yellow fever report |
| $X$ | Covariates used in the GLM for the presence/absence of any yellow fever report |
| $y$ | Observed presence or absence of yellow fever occurrence in the province |
| $b$ | Parameter anchoring the overall level of surveillance quality relative to the transmission intensity |
| $\theta$ | Per-case probability of detection of yellow fever |
| $n_{inf\_30, i}$ | Number of infections in province *i* over 30 year period |
| Demographic model | |
| $p(a,t)$ | Population proportion aged $a$ at time $t$ |
| $P(a,t)$ | Total number of individuals aged $a$ at time $t$ |
| $P_{tot}(t)$ | Total population number at time $t$ : $P_{tot}\left( t \right)=\sum_{a} P(a,t)$ |
| Force of infection model | |
| $\lambda$ | Force of infection |
| $v_{iat}$ | Vaccination coverage estimated in province *i* among individuals aged $a$ at time $t$ |
| $v_{eff}$ | Vaccine efficacy |
| $Prev(\lambda,u)$ | Expected seroprevalence over age group *u* assuming a force of infection equal to *λ* |
| R_0_ model | |
| $R_{0}$ | Basic reproduction number |
| $i^{-}v^{-}\left( a,t \right)$ | Proportion of susceptible individuals (previously uninfected, unvaccinated) aged $a$ at time $t$ |
| $i^{+}v^{-}\left( a,t \right)$ | Proportion of previously infected and unvaccinated individuals aged $a$ at time $t$ |
| $i^{-}v^{+}\left( a,t \right)$ | Proportion of previously uninfected and vaccinated individuals aged $a$ at time $t$ |
| $i^{+}v^{+}\left( a,t \right)$ | Proportion of previously infected and vaccinated individuals aged $a$ at time $t$ |
| $S\left( t \right)$ | Proportion of (previously uninfected, unvaccinated individuals) at time $t$ among all ages: $S\left( t \right)=\sum_{a} i^{-}v^{-}\left( a,t \right)p(a,t)$ |
| $\delta_{v}(a,t)$ | Incidence of vaccination among those aged $a$ at time $t$, accounting for imperfect  vaccine efficacy |
| $n_{inf}(a,t)$ | Number of newly infected at time $t$ among those aged $a$ |
| $N_{inf}(t)$ | Number of newly infected at time $t$ among all ages |
| ${Prev(R}_{0},u)$ | Expected seroprevalence over age group *u* assuming a basic reproduction number equal to $R_{0}$ |


## Generalized linear model for the presence/absence of any yellow fever report

Following the method described by Garske et al. [9], based on the dataset of yellow fever occurrence, a generalized linear model (GLM) was fitted to the presence of any yellow fever report at the province level, using as covariates the population size (log-transformed), the country-level surveillance quality metric as well as longitude, EVI and land cover type. This covariate set has previously been demonstrated to be the best set of predictors of the presence of any report of yellow fever at the province level [9]. As no surveillance quality metric was available for countries not covered by the YFSD, a country-level parameter was freely fitted.

A complementary log-log link function was used and the model prediction $q=q_{i}$ was given by:

|  | $q=1-exp(-e^{X\beta}),$ | (1) |
| --- | --- | --- |

where $X=\left( X_{ij} \right)$ is the matrix of covariates used in the model with $i$ indexing provinces, $j$ indexing the covariates, and $\beta=\beta_{j}$ is the parameter vector to be fitted.

The log-likelihood for this GLM can be written:

|  | $\ln L_{GLM}= \sum_{i} \left[ y_{i}\ln\left( q_{i} \right)+\left( 1-y_{i} \right)ln(1-q_{i}) \right],$ | (2) |
| --- | --- | --- |

assuming *y,* representing the presence or absence of yellow fever occurrence in the province $i$, is binomially distributed.

## Estimating the per-case probability of report

As described in Garske et al. [9], the GLM prediction, $q_{i}$, representing the probability of any yellow fever report at the province level over the 30-year period of interest, can be linked to the number of infections necessary to generate this probability, $n_{inf\_30, i}$ assuming a simple Poisson process for the detection of infection,

|  | $q_{i}=1-\left( 1-\theta_{i} \right)^{n_{inf\_30, i}}=1-exp[n_{inf\_30, i}\ln\left( 1-\theta_{i} \right)]$, | (3) |
| --- | --- | --- |

where $\theta_{i}=\theta_{c}$ is the per-case probability of detection of yellow fever, assumed to vary between, but not within, countries.

Using equation (1) $\theta_{i}$ can be identified as:

|  | $n_{inf\_30, i}\ln\left( 1-\theta_{i} \right)=-e^{{X_{i}\beta}_{i}}$. | (4) |
| --- | --- | --- |

By taking logarithms, this can be further transformed into:

|  | $\ln\left( n_{inf\_30, i} \right)={X_{i}\beta}_{i}-\beta_{c}-b_{i}$, | (5) |
| --- | --- | --- |

and

|  | $\ln\left( -\ln\left( 1-\theta_{c} \right) \right)= \beta_{c}+b,$ | (6) |
| --- | --- | --- |

where the parameter $b$ anchors the overall level of the surveillance quality relatively to the transmission intensity. Independent estimates of transmission intensity calculated in provinces covered by serological surveys were used to fit this parameter.

## Fitting serological data

Serological surveys were used to adjust the overall level of under-reporting. For the provinces covered by a serological survey, the number of infections over the study period can be estimated from the age-specific seroprevalence. This was done assuming two alternative models of transmission of yellow fever. In the static version of the model (thereafter termed “FOI model”, as a static force of infection is fitted) a constant, age-independent force of infection $\lambda$ is fitted to each serological survey. Alternatively, in the dynamical version of the model (thereafter termed “R_0_ model”), a basic reproduction number $R_{0}$ is fitted. One can consider the FOI model as a model assuming 100% of all transmission stemming from the environment (ie sporadic infections from the wildlife reservoir) whereas the R_0_ model assumes 100% of transmission stems from human-to-human transmission mediated by the anthropophilic vector of yellow fever (*Aedes aegypti*). As both transmission cycles contribute to the overall transmission of yellow fever, these models represent extreme transmission scenarios. The main difference between both versions of the model is that the FOI model only considers direct protection conferred by the yellow fever vaccine, whereas indirect benefits of vaccination due to herd immunity are captured in the R_0_ model.

Note that both versions of the model rely on the assumption that age-specific death rates are not affected by yellow fever infection or vaccination. Indeed, the yellow fever related mortality is already included in the overall mortality in the demographic projections used


### FOI model

Assuming a constant, age-independent force of infection, for each province $i$, the total number of infections over the 30-year period can be determined by the force of infection $\lambda_{i}$, the age- and time-specific population size $P(a,t)$ and the age- and time-dependant vaccination coverage $v(a,t)$, both of which also vary over time *t*:

|  | $n_{inf\_30, i}=\lambda_{i}\sum_{a, 1984\leq t\leq2013} e^{-\lambda_{i}a}P\left( a,t \right)\left( 1-v_{eff}v\left( a,t \right) \right) ,$ | (7) |
| --- | --- | --- |

where $v_{eff}$ is the vaccine efficacy.

Among provinces covered by a serological survey, for each age group *u* considered, the log-likelihood of $\lambda$, given the total number of sample tested $n_{tot, u}$ and the number of positive samples detected $n_{pos, u}$ is given by a binomial likelihood:

|  | $\ln L_{sero}= \sum_{u} ln\left[ \left( \begin{matrix} n_{tot, u} \\ n_{pos, u} \end{matrix} \right){Prev\left( \lambda,u \right)}^{n_{pos, u}}{(1-Prev\left( \lambda,u \right)}^{n_{tot, u}}) \right]$, | (8) |
| --- | --- | --- |

with $Prev\left( \lambda,u \right)$ being the expected seroprevalence over age group *u* assuming a force of infection equal to *λ.*

This version of the model has been previously described and used in [9].

### $R_{0}$ model

We developed an alternative, dynamic version of the model designed to include herd immunity, based on a SIR model with age structure. In this dynamic version, the value of $R_{0}$ is derived under the assumption of endemic equilibrium [33]. Further equations below (9 to 23) were implemented at the province level. However, the subscript , indexing provinces, is omitted for simplicity.

Endemic equilibrium is maintained by calculating the number of new infections such that the fraction of susceptible individuals in the population will equal $1/{R_{0}}$ in each year. This relationship can be expressed as:

|  | $N_{inf}\left( t \right)=max\left( 0,P_{tot}(t)\left( S\left( t \right)-\frac{1}{R_{0}} \right) \right)$, | (9) |
| --- | --- | --- |

with $S\left( t \right)$ and $P_{tot}(t)$ being the fraction of susceptibles and the total population size, respectively, and $N_{inf}\left( t \right)$ the number of infections required to maintain the endemic equilibrium in year $t$. Should the current fraction of susceptibles be lower than the threshold value for any year, no new infections are expected. In this model framework, such a situation can only arise due to vaccination activities. The main assumption for this model is that, for the whole study period the system is at endemic equilibrium, which leads to a constant value of $R_{0}$.

To properly account for the immunity expected during serological surveys for which vaccinated people are explicitly excluded, it is required that we keep track of both the infection and vaccination status of the population. Thus, four categories were created and denoted $i^{\pm}v^{\pm}$, where $i^{-}$ describes previously uninfected people, $i^{+}$ previously infected people, $v^{-}$ unvaccinated and $v^{+}$ vaccinated individuals.

As the four categories describe proportions, their sum equals 1:

|  | $i^{-}v^{-}\left( a,t \right)+i^{+}v^{-}\left( a,t \right)+i^{-}v^{+}\left( a,t \right)+i^{+}v^{+}\left( a,t \right)=1$ | (10) |
| --- | --- | --- |

For each province, the year of vaccine introduction $t_{0}$ was deduced from the vaccination database. We assumed there was no vaccination at all across Africa prior to $t_{0}=1940$ when the first yellow fever mass vaccination campaigns were implemented (thus all $t_{0}\geq1940$). In line with the assumption of the endemic equilibrium state, any infections arising during the pre-vaccination era ($t_{pre}<t_{0}$) simply serve to bring the proportion of susceptibles back down to the equilibrium solution for the given value of $R_{0}$, counterbalancing their replenishment through new births. While for an epidemic disease this might appear an unrealistic assumption, over the course of many years the higher incidence during epidemics balances the low incidence in inter-epidemic periods to achieve the equilibrium level on average.

For the pre-vaccination era ($t_{pre}<t_{0})$ we assumed that the force of infection does not depend on age $a$. The epidemic equilibrium assumption leads to a simple expression relating $R_{0}$ to the force of infection $\lambda$ and overall proportion of susceptibles $S(t_{pre})$:

|  | $S(t_{pre})$=$\frac{1}{R_{0}}\Longleftrightarrow R_{0}=\frac{1}{\sum_{a} i^{-}v^{-}\left( a,t_{pre} \right)p(a,t_{pre})}$ with $i^{-}v^{-}\left( a,t_{pre} \right)=e^{-\lambda a} .$ | (11) |
| --- | --- | --- |

For any given value of $R_{0}$, this can be solved numerically for $\lambda$ through Taylor expansion:

|  | $\sum_{a} p\left( a,t \right)\left[ 1-\lambda a+\frac{1}{2}\left( \lambda a \right)^{2}-\frac{1}{3!}\left( \lambda a \right)^{3}+\ldots\right]=\frac{1}{R_{0}}$ . | (12) |
| --- | --- | --- |

Based on the numerical solution for $\lambda$, the proportion susceptible in each age group $i^{-}v^{-}\left( a,t_{pre} \right)$ can be calculated according to (11).

At vaccination introduction, $t_{0}$, onwards, the annual number of new infections as well as vaccinations is explicitly tracked in order to take into account the effect vaccination has in reducing new infections. We assumed that the different events we considered follow the same sequence in each year $t$: aging occurs at $t+\tau_{1}$, new infections occur at $t+\tau_{2}$ and vaccination is implemented at $t+\tau_{3}$, with $\tau_{1}<\tau_{2}<\tau_{3}<1$ .

At the beginning of year $=t_{0}$ , the fraction of susceptibles $i^{-}v^{-}\left( a,t+\tau_{1} \right)$ equals that in the same cohort at the end of the previous year $t-1$, with newborns assumed susceptible:

|  | $i^{-}v^{-}\left( a,t+\tau_{1} \right)=i^{-}v^{-}\left( a-1,t-1+\tau_{3} \right)$, when $a>0$, | (13) |
| --- | --- | --- |
|  | $i^{-}v^{-}\left( 0,t+\tau_{1} \right)=1.$ |  |

As vaccination has not been implemented yet, we have:

|  | $i^{-}v^{+}\left( a,t+\tau_{1} \right)=i^{+}v^{+}\left( a,t+\tau_{1} \right)=0$, | (14) |
| --- | --- | --- |
|  | $i^{+}v^{-}\left( a,t+\tau_{1} \right)={1-i}^{-}v^{-}\left( a,t+\tau_{1} \right) .$ |  |

The number of new infections occurring at $t+\tau_{2}$ is calculated based on the fraction of susceptibles at $t+\tau_{1}$. From (13), we calculate $S\left( t+\tau_{1} \right)$ among all ages, which is used to determine the number of new infections needed in order to reach the endemic equilibrium:

|  | $N_{inf}\left( t \right)=max\left( 0,P_{tot}(t)\left( S\left( t+\tau_{1} \right)-\frac{1}{R_{0}} \right) \right) .$ | (15) |
| --- | --- | --- |

New infections are distributed across cohorts proportional to their contribution to the susceptible population:

|  | $n_{inf}\left( a,t \right)=N_{inf}\left( t \right)\frac{i^{-}v^{-}\left( a,t+\tau_{1} \right)p(a,t)}{\sum_{a} i^{-}v^{-}\left( a,t+\tau_{1} \right)} .$ | (16) |
| --- | --- | --- |

This number of new infections arises at time $t+\tau_{2}$ and reduces the number of susceptibles in the population:

|  | $i^{-}v^{-}\left( a,t+\tau_{2} \right)=i^{-}v^{-}\left( a,t+\tau_{1} \right)-\frac{n_{inf}\left( a,t \right)}{P\left( a,t \right)} ,$ $i^{+}v^{-}\left( a,t+\tau_{2} \right)=i^{+}v^{-}\left( a,t+\tau_{1} \right)+\frac{n_{inf}\left( a,t \right)}{P(a,t)} ,$ $i^{-}v^{+}\left( a,t+\tau_{2} \right)=i^{-}v^{+}\left( a,t+\tau_{1} \right) ,$ $i^{+}v^{+}\left( a,t+\tau_{2} \right)=i^{+}v^{+}\left( a,t+\tau_{1} \right) .$ | (17) |
| --- | --- | --- |

Finally, new vaccination is implemented at $t+\tau_{3}$. At that stage, one needs to be careful about the different categories, as through vaccination, the proportion of newly vaccinated is moved from the unvaccinated to the vaccinated categories, irrespective of infection status (those previously vaccinated that are re-vaccinated simply stay in their type and therefore cause no change):

|  | $i^{-}v^{-}\left( a,t+\tau_{3} \right)=i^{-}v^{-}\left( a,t+\tau_{2} \right)\left( 1-\delta_{v}\left( a,t \right) \right),$ $i^{+}v^{-}\left( a,t+\tau_{3} \right)=i^{+}v^{-}\left( a,t+\tau_{2} \right)\left( 1-\delta_{v}\left( a,t \right) \right),$ $i^{-}v^{+}\left( a,t+\tau_{3} \right)=i^{-}v^{+}\left( a,t+\tau_{2} \right)+i^{-}v^{-}\left( a,t+\tau_{2} \right)\delta_{v}\left( a,t \right),$ $i^{+}v^{+}\left( a,t+\tau_{3} \right)=i^{+}v^{+}\left( a,t+\tau_{2} \right)+i^{+}v^{-}\left( a,t+\tau_{2} \right)\delta_{v}\left( a,t \right),$ | (18) |
| --- | --- | --- |

where $\delta_{v}(a,t)$ is the vaccination incidence (*ie* the fraction of the cohort newly vaccinated in year $t$), already accounting for limited vaccine efficacy.

Then, for each year $t>t_{0}$, equations (13) to (18) apply similarly, with the exception of equation (14). Indeed, as vaccination has already been introduced, we now have:

|  | $i^{-}v^{-}\left( a,t+\tau_{1} \right)=i^{-}v^{-}\left( a-1,t-1+\tau_{3} \right)$ $i^{+}v^{-}\left( a,t+\tau_{1} \right)=i^{+}v^{-}\left( a-1,t-1+\tau_{3} \right)$,  $i^{-}v^{+}\left( a,t+\tau_{1} \right)= i^{-}v^{+}\left( a-1,t-1+\tau_{3} \right)$, $i^{+}v^{+}\left( a,t+\tau_{1} \right)= i^{+}v^{+}\left( a-1,t-1+\tau_{3} \right).$ | (19) |
| --- | --- | --- |
|  |  |  |

By tracking new infections arising each year, it is then possible to estimate the total number of infections arising over the 30-year study period:

|  | $n_{inf\_30}=\sum_{a, 1984\leq t\leq2013} n_{inf}\left( a,t \right).$ | (20) |
| --- | --- | --- |

It is also possible to calculate, for any value of $R_{0}$, the expected age-specific seroprevalence for all years. This seroprevalence among the total population is given by:

|  | $Prev\left( a,t \right)=1-i^{-}v^{-}\left( a,t \right)$, | (21) |
| --- | --- | --- |

whereas the prevalence among the unvaccinated population only is given by:

|  | ${Prev}^{v^{-}}\left( a,t \right)=1-\frac{i^{-}v^{-}\left( a,t \right)}{i^{-}v^{-}\left( a,t \right)+i^{+}v^{-}\left( a,t \right)}=\frac{i^{+}v^{-}\left( a,t \right)}{i^{-}v^{-}\left( a,t \right)+i^{+}v^{-}\left( a,t \right)} .$ . | (22) |
| --- | --- | --- |

These equations thus allow us to fit $R_{0}$ values to each serological survey, either for surveys including or excluding vaccinated people.

Similarly to equation (8), we assume that the number of samples testing positive in age group *u*, n_pos,u_, is binomially distributed given the total number of samples, n_tot,u_ . Therefore, the log-likelihood of *R_o_* is given by:

|  | $\ln L_{sero}= \sum_{u} ln\left[ \left( \begin{matrix} n_{tot, u} \\ n_{pos, u} \end{matrix} \right){Prev\left( R_{0},u \right)}^{n_{pos, u}}{(1-Prev\left( R_{0},u \right)}^{n_{tot, u}}) \right]$. | (23) |
| --- | --- | --- |

## Extrapolating the transmission parameters

For each serological survey, a transmission parameter is estimated (either λ or R0) and $n_{inf\_30, i}$ is calculated using equations (7) or (20). For each province covered by a serological survey, this number of infections over the 30-year study period is inserted into Equation (5). This calculation yields different estimates of the parameter $b_{i}$ for each province covered by a serological survey, therefore we calculate *b* as the mean of *b_i_*. We insert the estimate of *b* into equation (6) in order to calculate the country-specific, per-case detection probability, $\theta_{c}$; this then allows us to solve equation (5) to obtain $n_{inf\_30, i}$, but this time for all provinces and not just those covered by a serological survey. In the FOI model, $n_{inf\_30, i}$, is then inserted into equation (7) which is solved numerically for each value of $\lambda_{i}$. In the R0 model, $n_{inf\_30, i}$ is compared to a look-up table of infection numbers which is generated given a range of $R_{0}$ values for each province. The table infection numbers that are closest to $n_{inf\_30, i}$ give the interval of the $R_{0}$ in that province and we interpolate over this interval to obtain the final $R_{0}$ estimate.

## Estimating the total number of infections from transmission intensity

Using both versions of the model, the annual number of infections expected in any province $i$ for any year $t$ is estimated. In the FOI model, this is calculated using an equation similar to equation (7) for any specific value of $t$. In the R_0_ model, this is calculated using equations (11) to (16). This annual number of infections includes all infections, from asymptomatic infection to severe disease, potentially leading to death.

# Model fitting


In order to capture the uncertainty in resulting parameter estimates, all model parameters were estimated jointly using Monte Carlo Markov Chain (MCMC) simulations. The posterior distribution is therefore a function of the parameter prior distributions, log-likelihood from the GLM and log-likelihood from the serological studies given by either equation (8) or (23).

Non-informative priors were used for most of the model parameters, with the exception of vaccine efficacy. The prior distribution for this parameter is given by a logistic distribution informed by a meta-analysis of the available literature, with a point estimate for vaccine efficacy of 97.5% and a 95% credibility interval of 82.9 - 99.7% [25].

To improve the efficiency of our MCMC sampling, we update the gaussian proposal distribution for each parameter given the standard deviation of the MCMC chain for that parameter in exploratory runs. This is adjusted to arrive of an optimal acceptance ratio of between 0.2 and 0.4

Each version of the model was run for 1,000,000 iterations. Convergence was assessed visually. Additionally, the Raftery diagnostic was used to assess whether this number of iterations was sufficient to accurately estimate the quantiles of the parameters [50].Prior to further analysis, posterior samples were thinned by a factor of 1,000. Posterior samples were used to compute point estimates and 95% credibility intervals around model parameters as well as burden estimates.

# Burden estimates

The resulting posterior distribution estimates of the model parameters allows us to calculate the total number of infections. While disease transmission across the endemic region is estimated by the total number of infections, estimates of morbidity and mortality are more relevant in terms of population and health system.

In order to calculate the number of severe cases and deaths from all infections, we fitted log-normal distributions (truncated to values ≤1) to the point estimates and 95% credibility intervals of the proportion of severe diseases among all infections and proportions of death among severe infections estimated by Johansson et al [22]. For both distributions, samples were generated and multiplied by the total number of infections estimated by both versions of the model. This approach allows us to propagate the uncertainty surrounding the severity spectrum of the disease onto the total number of severe cases and deaths.

We also estimated the total number of Disability-adjusted life year lost (DALY) based on the number of years of life lost (YLL) and years lived with disabilities (YLD). The YLL were calculated from the previously calculated number of deaths stratified by age, year and country multiplied by the remaining life expectancy stratified equivalently. This was reconstituted from the demographic dataset by calculating, for each birth cohort in each country, the remaining life expectancy at age *a* conditional to surviving until age *a*. The number of YLD was calculated assuming a severe acute disease lasting on average *d_acute_* = 17.8 days among all severe cases [51], which was weighted with disability weights of *dw_acute_* = 0.172 [52]. We further assumed that the severe cases surviving the infection experienced a convalescent stage lasting around *d_convalescent_* = 28 days [51], weighted with disability weights of *dw_convalescent_* = 0.024 [52]. Long term disability caused by yellow fever infection is rare and was ignored in our YLD calculation. The durations and disability weights were assumed to be independent of age, and the overall YLDs are simply related to the total number of severe cases :

|  | $YLD=c(d_{acute}{dw}_{acute}+(1-CFR)d_{convalescent}{dw}_{convalescent)}$ | (24) |
| --- | --- | --- |

where *c* is the total number of severe cases and *CFR* is the case-fatality ratio.

# Vaccine impact estimates

We calculate the impact of any past or future vaccination activities by estimating the burden expected had these activities not taken place. The impact of any specific vaccination scenario was estimated by using the transmission parameters (either λ or R_0_) from the baseline model together with vaccination coverage levels for the scenario being considered in order to estimate the burden under that scenario. Considering the long-lasting (perhaps lifelong) effect of the yellow fever vaccine, we define the lifetime impact of vaccination as the cumulative difference over the 2000-2100 time period in baseline burden estimates and those estimated for the counterfactual scenario. Such a time horizon ensures to capture vaccine impact over most of the lifetime of people vaccinated and those benefitting from the resulting herd immunity.

Specifically, we estimate the impact of large massive vaccination campaigns conducted from 2006 under the Yellow Fever Initiative [53]. To that aim, the counterfactual vaccination scenario excluded all preventive vaccination campaigns conducted after 2005, but included post-2005 reactive vaccination campaigns and routine infant vaccination. After 2017, both baseline and counterfactual scenarios assumed constant levels of routine infant vaccination onwards at their 2015 levels.

**REFERENCES**

1. WHO. Yellow fever in Africa and the Americas, 2016. Wkly Epidemiol Rec WER. 2017;92: 442–452.

2. Disease Outbreak News. Yellow fever – Nigeria. Dis Outbreak News - WHO. 2017 [cited 9 Jan 2018]. Available: http://www.who.int/csr/don/22-december-2017-yellow-fever-nigeria/en/

3. Wang L, Zhou P, Fu X, Zheng Y, Huang S, Fang B, et al. Yellow fever virus: Increasing imported cases in China. J Infect. 2016;73: 377–380. doi:10.1016/j.jinf.2016.07.003

4. Nishino K, Yactayo S, Garcia E, Aramburu G, Manuel E, Costa A, et al. Yellow fever urban outbreak in Angola and the risk of extension. Wkly Epidemiol Rec WER. 2016;91: 181–192.

5. World Health Organization. Eliminate Yellow fever Epidemics (EYE): a global strategy, 2017–2026. Wkly Epidemiol Rec. 2017;92: 193–204.

6. Kraemer MUG, Faria NR, Reiner RC, Golding N, Nikolay B, Stasse S, et al. Spread of yellow fever virus outbreak in Angola and the Democratic Republic of the Congo 2015-16: a modelling study. Lancet Infect Dis. 2016. doi:10.1016/S1473-3099(16)30513-8

7. Zhao S, Stone L, Gao D, He D. Modelling the large-scale yellow fever outbreak in Luanda, Angola, and the impact of vaccination. PLoS Negl Trop Dis. 2018;12: e0006158. doi:10.1371/journal.pntd.0006158

8. Rogers DJ, Wilson AJ, Hay SI, Graham AJ. The global distribution of yellow fever and dengue. Adv Parasitol. 2006;62: 181–220. doi:10.1016/S0065-308X(05)62006-4

9. Garske T, Van Kerkhove MD, Yactayo S, Ronveaux O, Lewis RF, Staples JE, et al. Yellow Fever in Africa: Estimating the Burden of Disease and Impact of Mass Vaccination from Outbreak and Serological Data. PLoS Med. 2014;11: e1001638. doi:10.1371/journal.pmed.1001638

10. Shearer FM, Longbottom J, Browne AJ, Pigott DM, Brady OJ, Kraemer MUG, et al. Existing and potential infection risk zones of yellow fever worldwide: a modelling analysis. Lancet Glob Health. 2018;0. doi:10.1016/S2214-109X(18)30024-X

11. World Health Organization. Yellow fever key facts. 1 May 2018. Available: http://www.who.int/en/news-room/fact-sheets/detail/yellow-fever

12. Faria NR, Kraemer MUG, Hill SC, Jesus JG de, Aguiar RS, Iani FCM, et al. Genomic and epidemiological monitoring of yellow fever virus transmission potential. Science. 2018; eaat7115. doi:10.1126/science.aat7115

13. Monath TP, Vasconcelos PFC. Yellow fever. J Clin Virol. 2015;64: 160–173. doi:10.1016/j.jcv.2014.08.030

14. Hamlet A, Jean K, Yactayo S, Benzler J, Cibrelus L, Ferguson N, et al. POLICI: A web application for visualising and extracting yellow fever vaccination coverage in Africa. Vaccine. 2019;37: 1384–1388. doi:10.1016/j.vaccine.2019.01.074

15. WHO, UNICEF. Yellow Fever Initiative Joint WHO and UNICEF 2010 Progress Report. Geneva, Switzerland: World Health Organisation; 2010.

16. Bhatt S, Gething PW, Brady OJ, Messina JP, Farlow AW, Moyes CL, et al. The global distribution and burden of dengue. Nature. 2013;496: 504–507. doi:10.1038/nature12060

17. World Health Organization. Yellow fever vaccine. Wkly Epidemiol Rec. 2003; 349–359.

18. Kwagonza L, Masiira B, Kyobe-Bosa H, Kadobera D, Atuheire EB, Lubwama B, et al. Outbreak of yellow fever in central and southwestern Uganda, February-may 2016. BMC Infect Dis. 2018;18: 548. doi:10.1186/s12879-018-3440-y

19. Bagcchi S. Looking back at yellow fever in Angola. Lancet Infect Dis. 2017;17: 269–270. doi:10.1016/S1473-3099(17)30064-6

20. Gaythorpe KAM, Jean K, Cibrelus L, Garske T. Quantifying model evidence for yellow fever transmission routes in Africa. PLOS Comput Biol. 2019;15: e1007355. doi:10.1371/journal.pcbi.1007355

21. Robertson SE, Hull BP, Tomori O, Bele O, LeDuc JW, Esteves K. Yellow fever: a decade of reemergence. JAMA. 1996;276: 1157–1162.

22. Johansson MA, Vasconcelos PFC, Staples JE. The whole iceberg: estimating the incidence of yellow fever virus infection from the number of severe cases. Trans R Soc Trop Med Hyg. 2014;108: 482–487. doi:10.1093/trstmh/tru092

23. Fine P, Eames K, Heymann DL. “Herd immunity”: a rough guide. Clin Infect Dis Off Publ Infect Dis Soc Am. 2011;52: 911–916. doi:10.1093/cid/cir007

24. Shearer FM, Moyes CL, Pigott DM, Brady OJ, Marinho F, Deshpande A, et al. Global yellow fever vaccination coverage from 1970 to 2016: an adjusted retrospective analysis. Lancet Infect Dis. 2017;0. doi:10.1016/S1473-3099(17)30419-X

25. Jean K, Donnelly CA, Ferguson NM, Garske T. A Meta-Analysis of Serological Response Associated with Yellow Fever Vaccination. Am J Trop Med Hyg. 2016;95: 1435–1439. doi:10.4269/ajtmh.16-0401

26. World Health Organization. Yellow fever in Africa and South America, 2015. Wkly Epidemiol Rec. 2016;91: 381–388.

27. Hamlet A, Jean K, Perea W, Yactayo S, Biey J, Kerkhove MV, et al. The seasonal influence of climate and environment on yellow fever transmission across Africa. PLoS Negl Trop Dis. 2018;12: e0006284. doi:10.1371/journal.pntd.0006284

28. Kraemer MUG, Sinka ME, Duda KA, Mylne AQN, Shearer FM, Barker CM, et al. The global distribution of the arbovirus vectors Aedes aegypti and Ae. albopictus. eLife. 2015;4: e08347. doi:10.7554/eLife.08347

29. de Almeida MAB, dos Santos E, Cardoso J da C, da Silva LG, Rabelo RM, Bicca-Marques JC. Predicting Yellow Fever Through Species Distribution Modeling of Virus, Vector, and Monkeys. EcoHealth. 2018 [cited 7 Jan 2019]. doi:10.1007/s10393-018-1388-4

30. Vos T, Allen C, Arora M, Barber RM, Bhutta ZA, Brown A, et al. Global, regional, and national incidence, prevalence, and years lived with disability for 310 diseases and injuries, 1990–2015: a systematic analysis for the Global Burden of Disease Study 2015. The Lancet. 2016;388: 1545–1602. doi:10.1016/S0140-6736(16)31678-6

31. Monath TP, Woodall JP, Gubler DJ, Yuill TM, Mackenzie JS, Martins RM, et al. Yellow fever vaccine supply: a possible solution. The Lancet. 2016;387: 1599–1600. doi:10.1016/S0140-6736(16)30195-7

32. Global Strategy to Eliminate Yellow fever Epidemics (EYE) - Document for SAGE. World Health Organisation; 2016 Sep. Available: http://www.who.int/immunization/sage/meetings/2016/october/2_EYE_Strategy.pdf

33. Anderson R, May RM. Infectious Diseases of Humans: Dynamics and Control. 2nd edn. Oxford University Press; 1991.

34. Gotuzzo E, Yactayo S, Córdova E. Efficacy and duration of immunity after yellow fever vaccination: systematic review on the need for a booster every 10 years. Am J Trop Med Hyg. 2013;89: 434–444. doi:10.4269/ajtmh.13-0264

35. United Nations, Department of Economic and Social Affairs, Population Division, Population Estimates and Projections Section. World Population Prospects, the 2015 Revision. 2015 [cited 14 Jul 2016]. Available: https://esa.un.org/unpd/wpp/

36. Garske T, Ferguson NM, Ghani AC. Estimating Air Temperature and Its Influence on Malaria Transmission across Africa. PLoS ONE. 2013;8: e56487. doi:10.1371/journal.pone.0056487

37. Dobson JE, Bright EA, Coleman PR, Durfee RC, Worley BA. LandScan: A global population database for estimating populations at risk. Photogramm Eng Remote Sens. 2000;66: 849–857.

38. Durieux C. Mass yellow fever vaccination in French Africa south of the Sahara. Geneva: WHO.; 1956 pp. 115–122.

39. WHO, UNICEF. A Strategic Framework for the Yellow Fever Immunization Initiative 2012-2020: Beyond the Investment Case in Africa. 2012.

40. WHO/UNICEF Estimates of National Immunization Coverage (WUENIC). In: WHO-UNICEF estimates of YFV coverage [Internet]. 2015 [cited 14 Jul 2016]. Available: http://apps.who.int/immunization_monitoring/globalsummary/timeseries/tswucoverageyfv.html

41. Diallo M, Janusz K, Lewis RF, Manengu C, Sall A, Staples JE, et al. Rapid Assessment of Yellow Fever Viral Activity in the Central African Republic. World Health Organization; 2010.

42. Kuniholm MH, Wolfe ND, Huang CY-H, Mpoudi-Ngole E, Tamoufe U, LeBreton M, et al. Seroprevalence and distribution of Flaviviridae, Togaviridae, and Bunyaviridae arboviral infections in rural Cameroonian adults. Am J Trop Med Hyg. 2006;74: 1078–1083.

43. Merlin M, Josse R, Kouka-Bemba D, Meunier D, Senga J, Simonkovich E, et al. [Evaluation of immunological and entomotological indices of yellow fever in Pointe-Noire, People’s Republic of Congo]. Bull Société Pathol Exot Ses Fil. 1986;79: 199–206.

44. Omilabu SA, Adejumo JO, Olaleye OD, Fagbami AH, Baba SS. Yellow fever haemagglutination-inhibiting, neutralising and IgM antibodies in vaccinated and unvaccinated residents of Ibadan, Nigeria. Comp Immunol Microbiol Infect Dis. 1990;13: 95–100.

45. Tsai TF, Lazuick JS, Ngah RW, Mafiamba PC, Quincke G, Monath TP. Investigation of a possible yellow fever epidemic and serosurvey for flavivirus infections in northern Cameroon, 1984. Bull World Health Organ. 1987;65: 855–860.

46. Werner GT, Huber HC, Fresenius K. [Prevalence of yellow fever antibodies in north Zaire]. Ann Société Belge Médecine Trop. 1985;65: 91–93.

47. NASA Land Processes Distributed Active Archive Center (LP DAAC). Vegetation Indices 16-Day L3 Global 1 km (13A2). Sioux Falls, South Dakota: USGS/Earth Resources Observation and Science (EROS) Center,; Available: http://lpdaac.usgs.gov/get_data

48. Loveland TR, Belward AS. The IGBP-DIS global 1km land cover data set, DISCover: First results. Int J Remote Sens. 1997;18: 3289–3295. doi:10.1080/014311697217099

49. Global Administrative Areas | Boundaries without limits. [cited 20 Sep 2016]. Available: http://www.gadm.org/

50. Raffery A, Lewis S. One long run with diagnostics: Implementation strategies for Markov chain Monte Carlo. Stat Sci. 1992; 493:497.

51. Monath TP. Yellow fever: an update. Lancet Infect Dis. 2001;1: 11–20. doi:10.1016/S1473-3099(01)00016-0

52. LaBeaud Ad, Bashir F, King CH. Measuring the burden of arboviral diseases: the spectrum of morbidity and mortality from four prevalent infections. Popul Health Metr. 2011;9: 1. doi:10.1186/1478-7954-9-1

53. Yellow Fever Initiative: Poviding an opportunity of a lifetime. World Health Organization; 2010. Available: www.who.int/csr/disease/yellowfev/YFIbrochure.pdf
