# Supplementary material for: Eliminating yellow fever epidemics in Africa: Vaccine demand forecast and impact modelling
Source: PLoS Negl Trop Dis. 2020 May 7;14(5):e0008304. doi: 10.1371/journal.pntd.0008304 (PMC7237041; doi:10.1371/journal.pntd.0008304)
Supplement: S2 Appendix — (DOCX) [file pntd.0008304.s003.docx]

**Eliminating yellow fever epidemics in Africa: vaccine demand forecast and impact modelling**

**Short title :** Modelling the Elimination of Yellow Fever epidemics in Africa

**S2 Appendix : Model validation**

Kévin Jean^1,2,3^*, Arran Hamlet^3^, Justus Benzler^4,5^, Laurence Cibrelus^4^, Katy A. M. Gaythorpe^3^, Amadou Sall^6^, Neil M. Ferguson^3^, Tini Garske^3^.

1. Laboratoire MESuRS, Conservatoire National des Arts et Métiers, Paris, France
2. Unité PACRI, Institut Pasteur, Conservatoire National des Arts et Métiers, Paris, France
3. MRC Centre for Global Infectious Disease Analysis, Department of Infectious Disease Epidemiology, Imperial College, London, UK
4. Infectious Hazard Management, World Health Organization, Geneva, Switzerland.
5. Robert Koch Institut, Berlin, Germany
6. Arbovirus and viral haemorrhagic fever unit, Institut Pasteur de Dakar, Dakar, Senegal

* Correspondance to:

Kévin Jean

Laboratoire MESuRS, Conservatoire National des Arts et Métiers, 292 rue Saint Martin, 75003, Paris, France

kevin.jean@lecnam.net

Model predictions were compared to recent results of serological surveys conducted in the Democratic Republic of Congo, South Sudan and Chad. Results of these surveys were shared as age-seroprevalence profiles aggregated by ecological zone (regions sharing similar ecological characteristics, as defined by national authorities), with one or more survey sites per ecological zone. Based on the localisation of the survey sites, for each ecological zone, the results were attributed to one or several provinces.

Validation was conducted firstly by comparing transmission intensity parameters (FOI or R_0_) estimated by fitting the age-seroprevalence profiles to those predicted in the corresponding provinces by the Yellow Fever burden model (main text, Figure 3). This method presents the advantage to produce estimated and predicted transmission parameters together with 95% credibility intervals that can be easily compared. However, the estimation of the transmission parameters based on the age-seroprevalence profiles already results from model fitting.

We thus completed this approach by directly comparing age-seroprevalence profiles observed in the surveys to those predicted by both variants of the Yellow Fever burden model. Results are presented in Figures 1 to 3 of this supplementary text. We compared the predictions of each model variants to the observed age-seroprevalence profiles using the likelihood criteria (same number of parameters for both model variants) (Table 1).


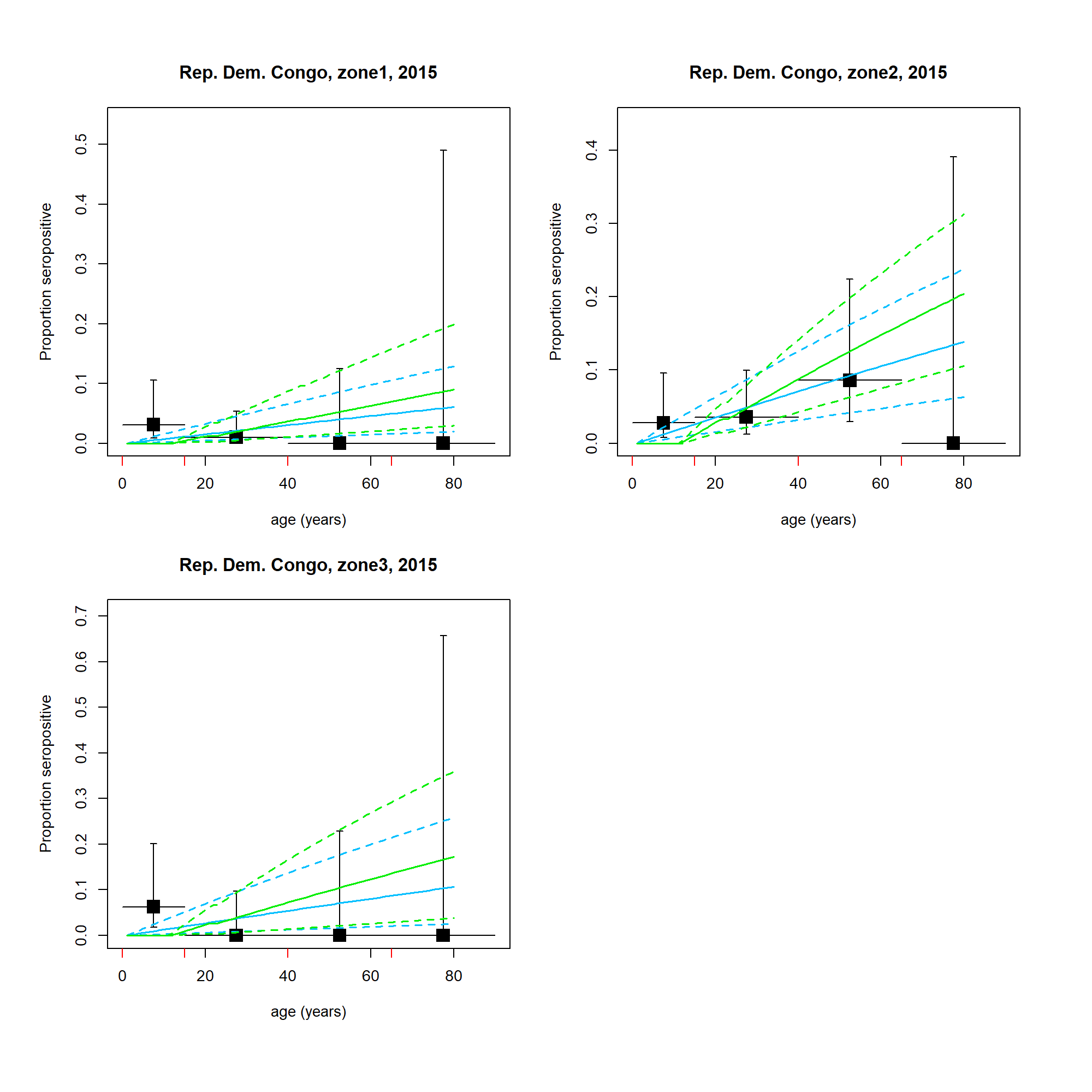


**Appendix 2 – Figure 1: Comparison of seroprevalence observed in 2015 in the Democratic Republic of Congo and predictions of the FOI and R_0_ models.** Black dots: observed data; vertical black line: 95% confidence interval for observed data. Blue: FOI model; Green: R0 model; solid line: median prediction; dashed coloured lines: 95% credibility intervals around predictions.


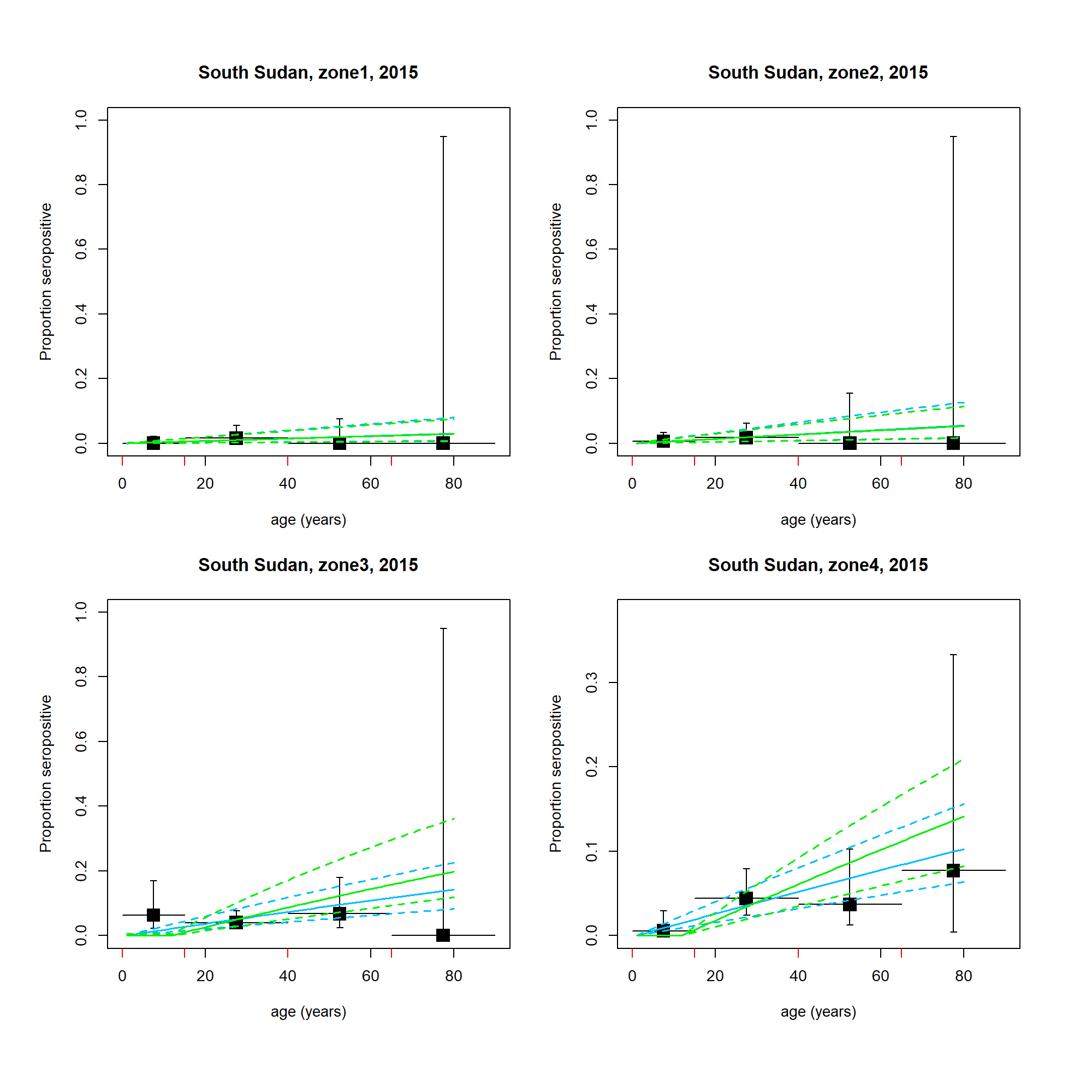


**Appendix 2 – Figure 2: Comparison of seroprevalence observed in 2015 in South Sudan and predictions of the FOI and R_0_ models.** Black dots: observed data; vertical black line: 95% confidence interval for observed data. Blue: FOI model; Green: R0 model; solid line: median prediction; dashed coloured lines: 95% credibility intervals around predictions.


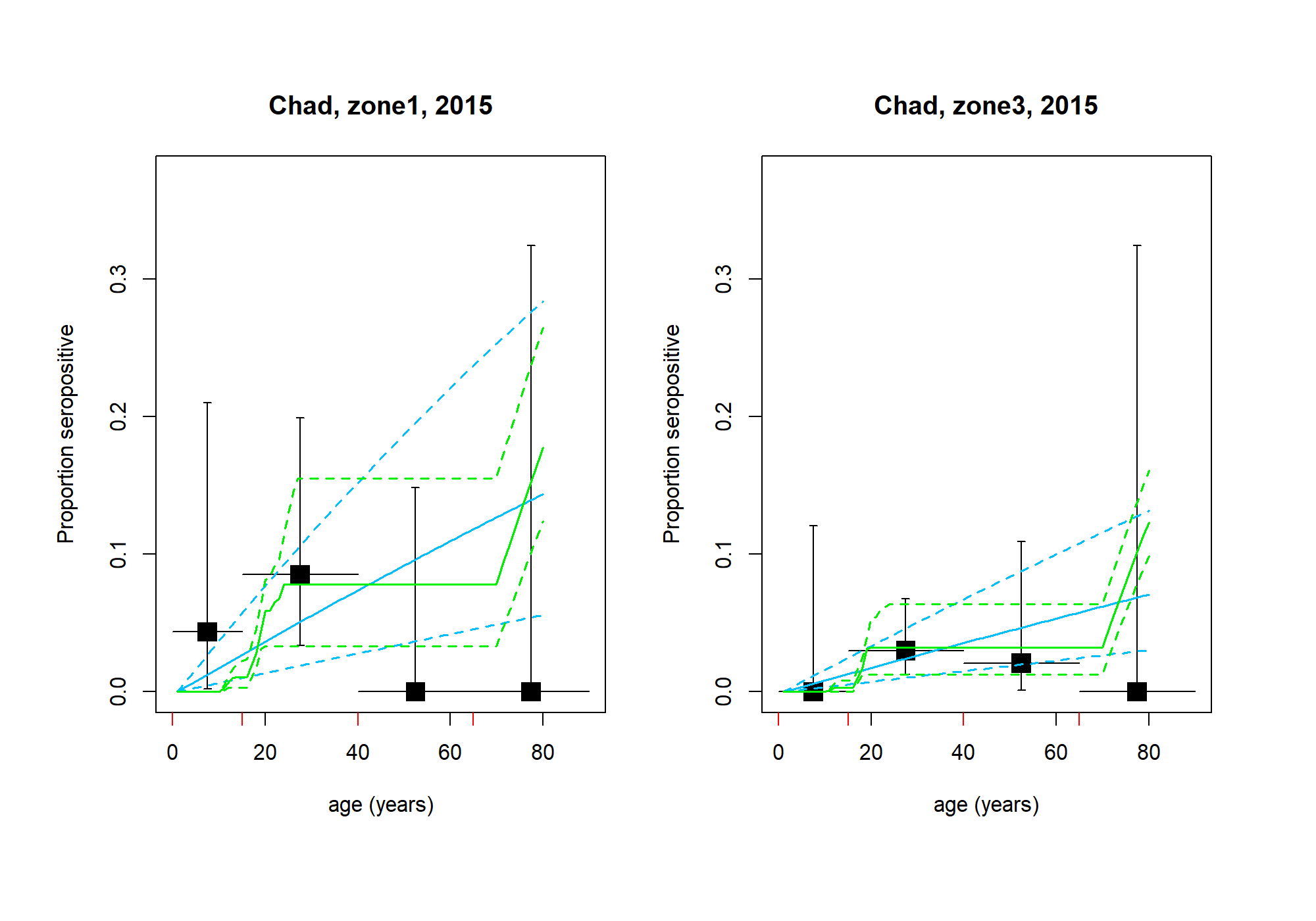


**Appendix 2 – Figure 3: Comparison of seroprevalence observed in 2015 in Chad and predictions of the FOI and R_0_ models.** Black dots: observed data; vertical black line: 95% confidence interval for observed data. Blue: FOI model; Green: R0 model; solid line: median prediction; dashed coloured lines: 95% credibility intervals around predictions.

**Appendix 2 – Table 1: Comparison of both FOI and R_0_ models’ prediction to observed age-seroprevalence profiles in 9 survey settings seroprevalence.** As the number of parameters is the same in both model versions, comparison is based on the likelihood criteria. DRC : Democratic Republic of the Congo.

| **Study setting** | **FOI model log-likelihood** | **R0 model loglikelihood** |
| --- | --- | --- |
| DRC, zone 1, 2015 | -5.87756930 | -10.7327074 |
| DRC, zone 2, 2015 | -5.71764888 | -9.90969063 |
| DRC, zone 3, 2015 | -5.90418027 | -10.5876885 |
| South Sudan, zone 1, 2015 | -2.87311369 | -2.89984104 |
| South Sudan, zone 2, 2015 | -3.12118973 | -3.08282078 |
| South Sudan, zone 3, 2015 | -7.87374162 | -15.0911081 |
| South Sudan, zone 4, 2015 | -6.66551947 | -8.74720144 |
| Chad, zone1, 2015 | -7.01385991 | -7.58303835 |
| Chad, zone3, 2015 | -3.99322086 | -3.40960100 |
