# Supplementary material for: Eliminating yellow fever epidemics in Africa: Vaccine demand forecast and impact modelling
Source: PLoS Negl Trop Dis. 2020 May 7;14(5):e0008304. doi: 10.1371/journal.pntd.0008304 (PMC7237041; doi:10.1371/journal.pntd.0008304)
Supplement: S1 Fig — A: Presence (red) or absence (white) of YF report between 1984 and 2013, B: GLM prediction for probability of report for the FOI model; C: GLM prediction for probability of report for the R0 model. GLM: Generalized Linear Model. Maps were produced from GADM version 2.0. (DOCX) [file pntd.0008304.s004.docx]

**Eliminating yellow fever epidemics in Africa: vaccine demand forecast and impact modelling**

**Short title :** Modelling the Elimination of Yellow Fever epidemics in Africa

**S1 Figure**


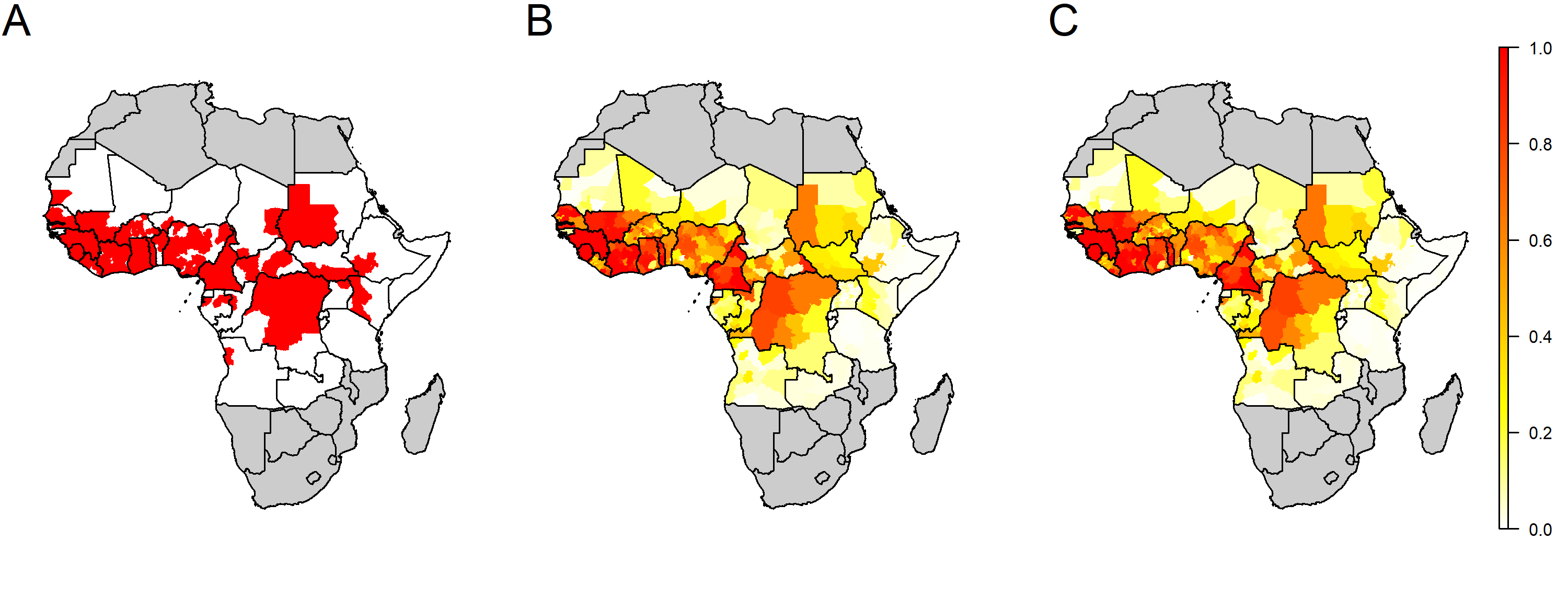


**S1 Figure: Observed versus predicted presence/absence of any yellow fever reported event between 1984 and 2013.** A: Presence (red) or absence (white) of YF report between 1984 and 2013, B: GLM prediction for probability of report for the FOI model; C: GLM prediction for probability of report for the R0 model. Maps were produced from GADM version 2.0.

GLM: Generalized Linear Model.
