# Supplementary material for: Eliminating yellow fever epidemics in Africa: Vaccine demand forecast and impact modelling
Source: PLoS Negl Trop Dis. 2020 May 7;14(5):e0008304. doi: 10.1371/journal.pntd.0008304 (PMC7237041; doi:10.1371/journal.pntd.0008304)
Supplement: S2 Fig — A: FOI model, B: R0 model. Maps were produced from GADM version 2.0. (DOCX) [file pntd.0008304.s005.docx]

**Eliminating yellow fever epidemics in Africa: vaccine demand forecast and impact modelling**

**Short title :** Modelling the Elimination of Yellow Fever epidemics in Africa

**S2 Figure**


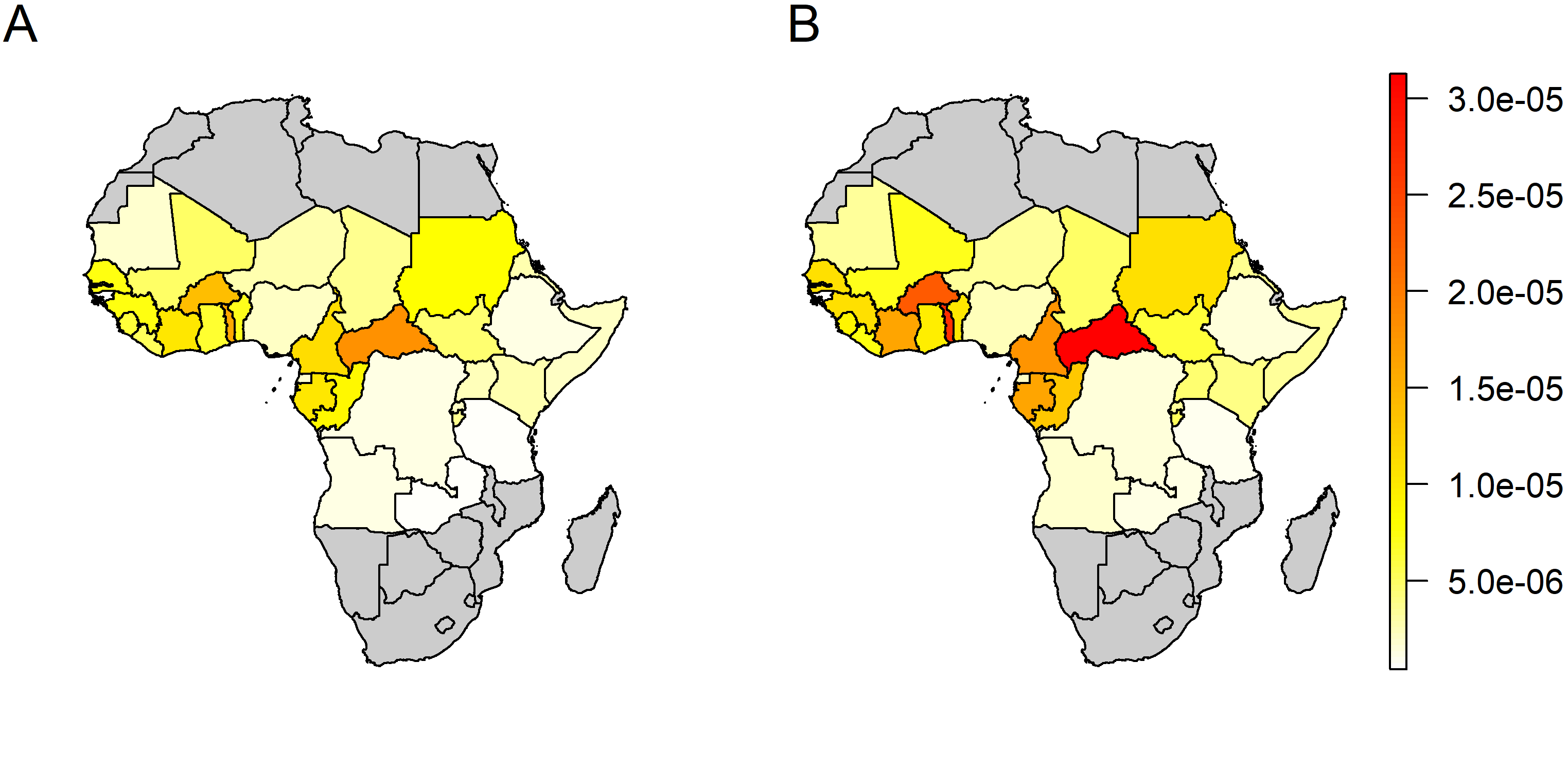


**S2 Figure: Country-specific per-infection probability of detection across both model variants.** A: FOI model, B: R_0_ model. Maps were produced from GADM version 2.0.
