# Supplementary material for: Eliminating yellow fever epidemics in Africa: Vaccine demand forecast and impact modelling
Source: PLoS Negl Trop Dis. 2020 May 7;14(5):e0008304. doi: 10.1371/journal.pntd.0008304 (PMC7237041; doi:10.1371/journal.pntd.0008304)
Supplement: S3 Fig — A: coefficient of variation of the force of infection estimates; B: coefficient of variation of the R0 estimates. Maps were produced from GADM version 2.0. (DOCX) [file pntd.0008304.s006.docx]

**Eliminating yellow fever epidemics in Africa: vaccine demand forecast and impact modelling**

**Short title :** Modelling the Elimination of Yellow Fever epidemics in Africa

**S3 Figure**


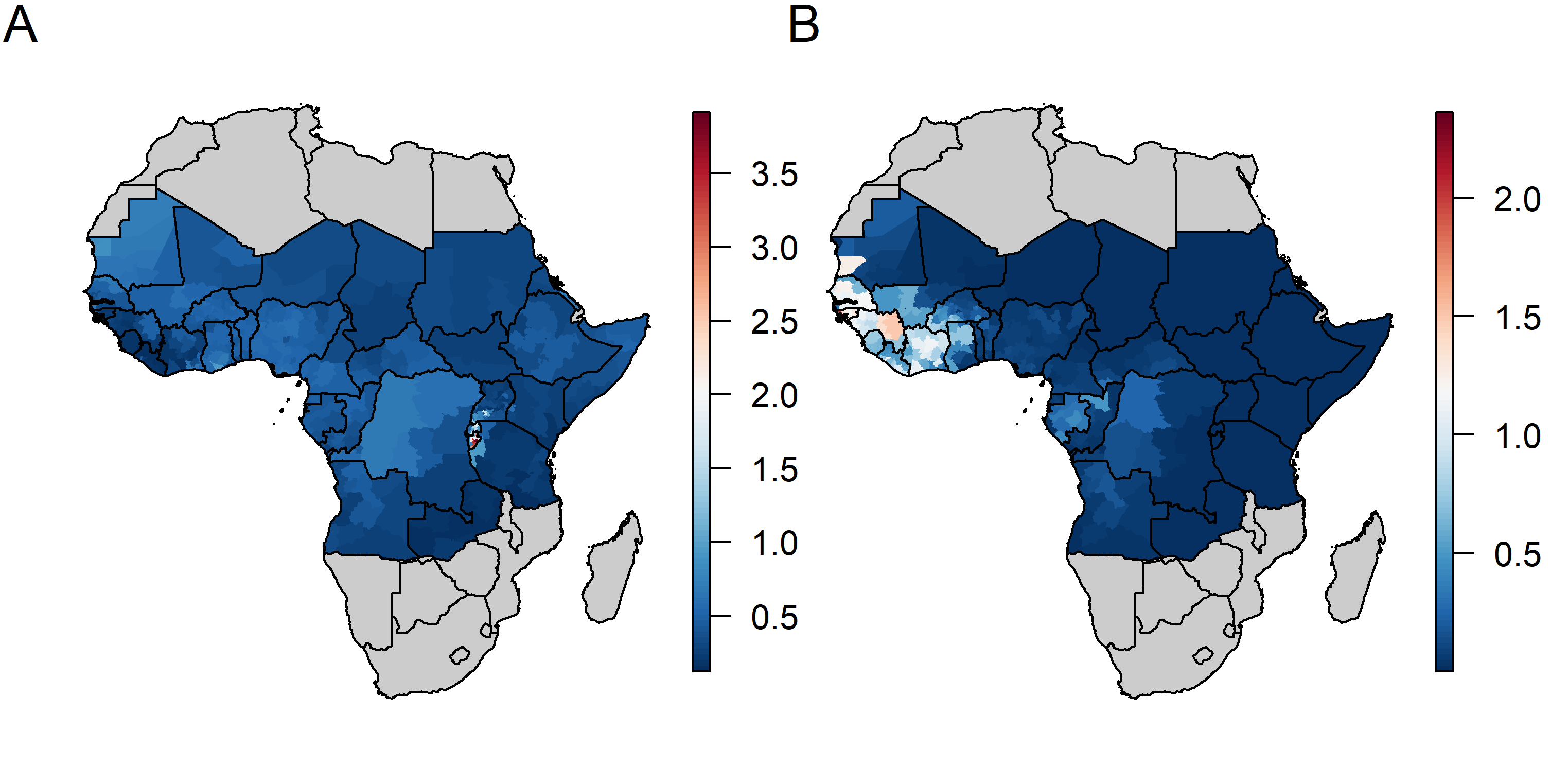


**S3 Figure: Variability in the estimates of transmission intensity across model variants.** A: coefficient of variation of the force of infection estimates; B: coefficient of variation of the R_0_ estimates. Maps were produced from GADM version 2.0.
