# Supplementary material for: Eliminating yellow fever epidemics in Africa: Vaccine demand forecast and impact modelling
Source: PLoS Negl Trop Dis. 2020 May 7;14(5):e0008304. doi: 10.1371/journal.pntd.0008304 (PMC7237041; doi:10.1371/journal.pntd.0008304)
Supplement: S4 Fig — R0 model. Maps were produced from GADM version 2.0. (DOCX) [file pntd.0008304.s007.docx]

**Eliminating yellow fever epidemics in Africa: vaccine demand forecast and impact modelling**

**Short title :** Modelling the Elimination of Yellow Fever epidemics in Africa

**S4 – Figure**


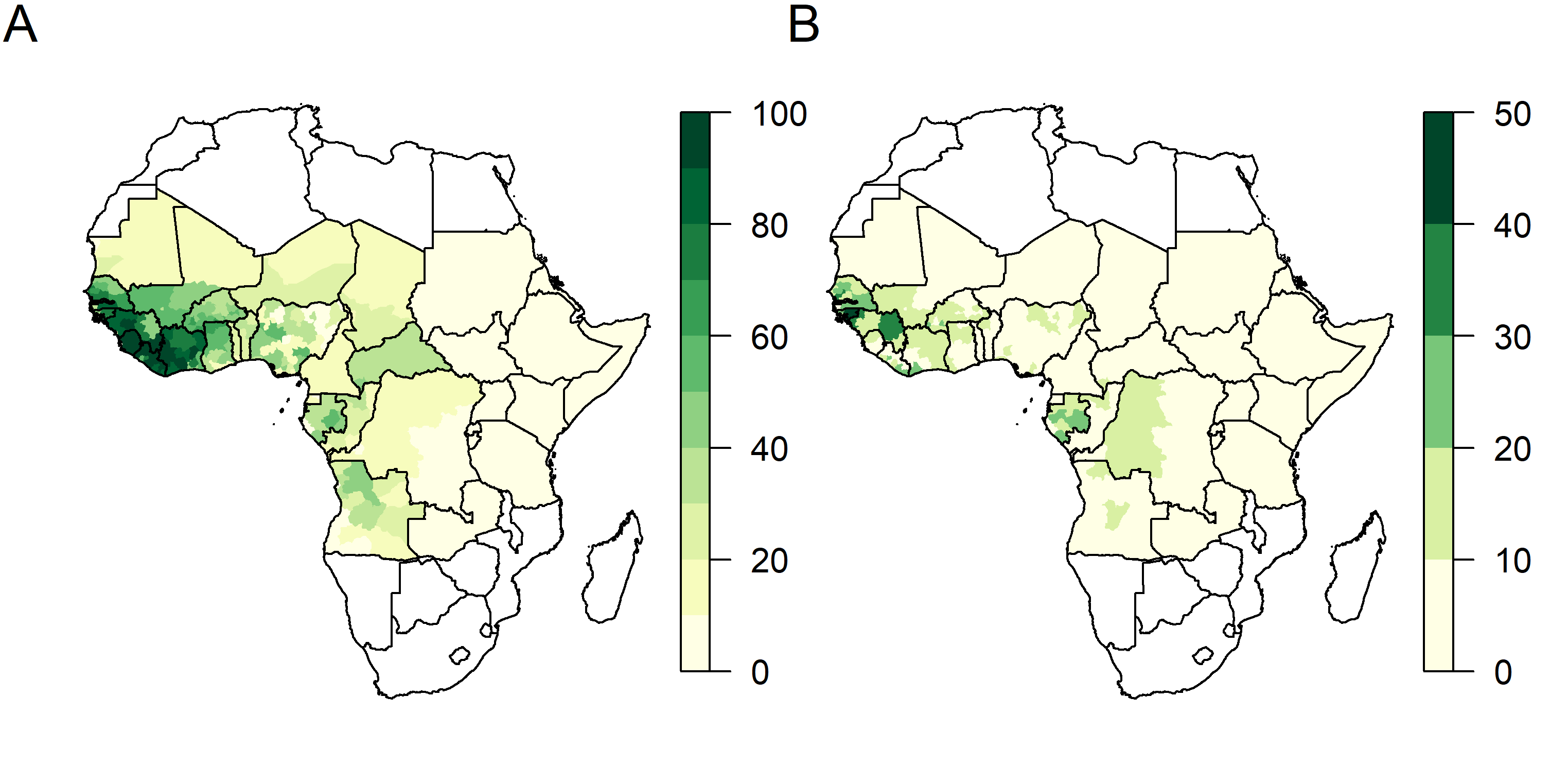


**S4 Figure : Estimated median critical vaccination coverage (A, %) and interquartile range in the critical vaccination coverage (B, %).** R0 model. Maps were produced from GADM version 2.0.
