# Supplementary material for: Eliminating yellow fever epidemics in Africa: Vaccine demand forecast and impact modelling
Source: PLoS Negl Trop Dis. 2020 May 7;14(5):e0008304. doi: 10.1371/journal.pntd.0008304 (PMC7237041; doi:10.1371/journal.pntd.0008304)
Supplement: S5 Fig — Light blue lines: individual FOI model runs, 1,000 simulations. Dark blue line: median FOI model burden. Blue region: 95% Credible Interval of the FOI model burden. Light green lines: individual R0 model runs, 1,000 simulations. Dark green line: median R0 model burden. Green region: 95% Credible Interval of the FOI model burden. Grey line: overall population-level vaccination coverage across the whole endemic region. (DOCX) [file pntd.0008304.s008.docx]

**Eliminating yellow fever epidemics in Africa: vaccine demand forecast and impact modelling**

**Short title :** Modelling the Elimination of Yellow Fever epidemics in Africa

**S5 Figure**

***
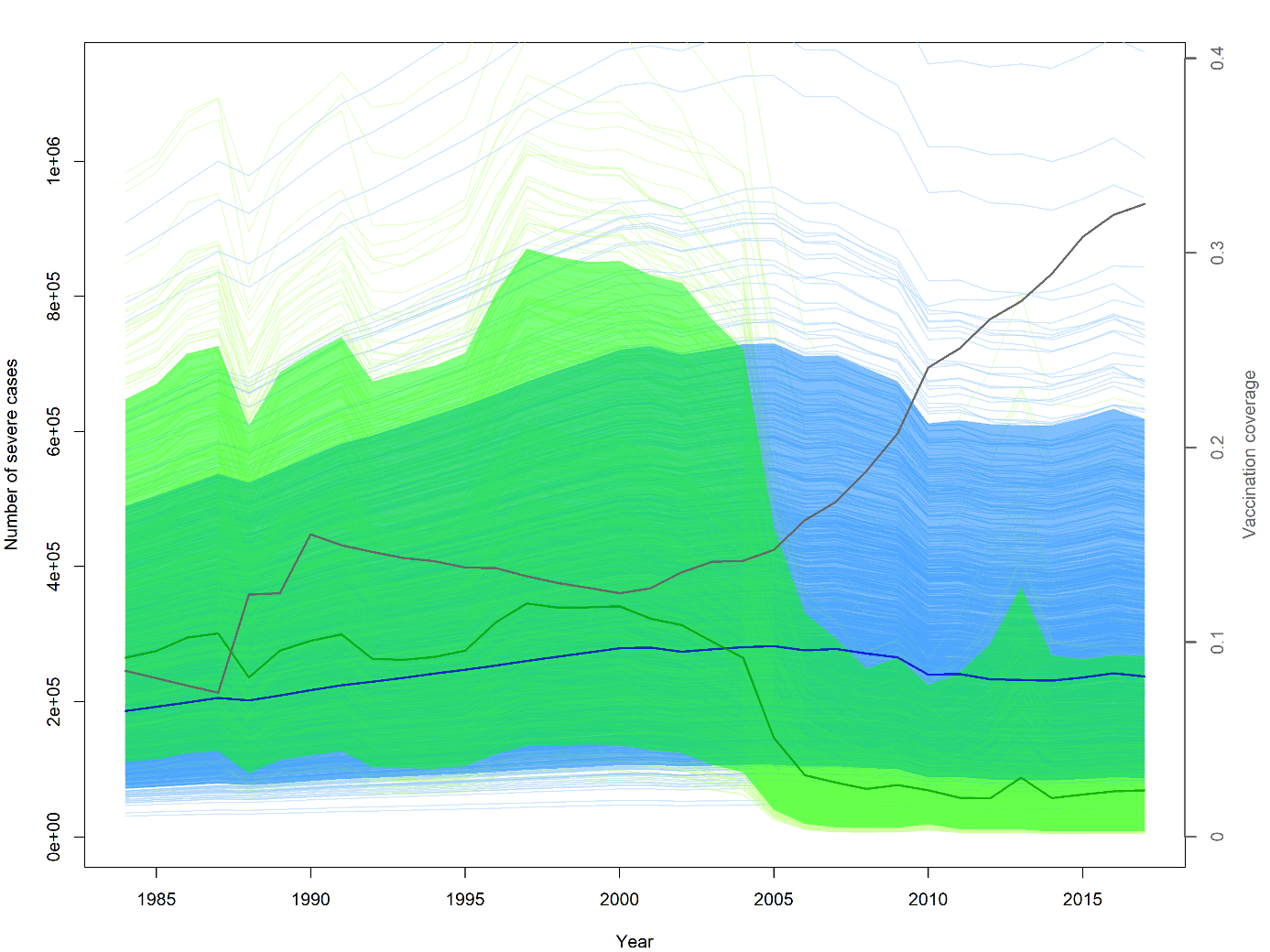
***

**S5 Figure: Comparison of yellow fever burden estimates over time between both model versions across the whole endemic region (34 countries).** Light blue lines: individual FOI model runs, 1,000 simulations. Dark blue line: median FOI model burden. Blue region: 95% Credible Interval of the FOI model burden. Light green lines: individual R0 model runs, 1,000 simulations. Dark green line: median R0 model burden. Green region: 95% Credible Interval of the FOI model burden. Grey line: overall population-level vaccination coverage across the whole endemic region.
